# Hypoxia-Related Marker GLUT-1, CAIX, Proliferative Index and Microvessel Density in Canine Oral Malignant Neoplasia

**DOI:** 10.1371/journal.pone.0149993

**Published:** 2016-02-23

**Authors:** Valeria Meier, Franco Guscetti, Malgorzata Roos, Stefanie Ohlerth, Martin Pruschy, Carla Rohrer Bley

**Affiliations:** 1 Division of Radiation Oncology, Vetsuisse Faculty University of Zurich, Zurich, Switzerland; 2 Institute of Veterinary Pathology, University of Zurich, Zurich, Switzerland; 3 Department of Biostatistics, Epidemiology Biostatistics and Prevention Institute, Faculty of Medicine, University of Zurich, Zurich, Switzerland; 4 Clinic of Diagnostic Imaging, Vetsuisse Faculty University of Zurich, Zurich, Switzerland; 5 Laboratory for Molecular Radiobiology, Radiation Oncology, University Hospital Zurich, Zurich, Switzerland; Colorado State University, UNITED STATES

## Abstract

For various types of tumor therapy, it is suggested that co-targeting of tumor microenvironment, mainly tumor vasculature, mediates tumor response mechanisms. Immunohistochemistry for glucose transporter-1 (GLUT-1), carbonic anhydrase-IX (CAIX), Ki-67, and von Willebrand factor VIII for microvessel density (MVD) were performed on formalin-fixed paraffin-embedded samples of canine oral malignant neoplasms. Polarographic oxygen measurements (median pO2) and perfusion data via contrast-enhanced power Doppler ultrasound (median vascularity, median blood volume) provided additional information. Ninety-two samples were analyzed: sarcomas (n = 32), carcinomas (n = 30), and malignant melanomas (n = 30). Polarographic oxygen and perfusion data was available in 22.8% (sarcomas n = 9, carcinomas n = 7, melanomas n = 5), and 27.1% (sarcomas n = 10, carcinomas n = 8, melanomas n = 7) of cases, respectively. GLUT-1 expression was detected in 46.7% of all samples, and was generally weak. CAIX expression was found in 34.8% of all samples. Median Ki-67 score and MVD count was 19% and 17, respectively. The evaluation of the GLUT-1 score and continuous data showed significantly lower GLUT-1 levels in sarcomas (mean 5.1%, SD 6.2) versus carcinomas and melanomas (mean 16.5%/ 19.0%, SD 17.3/ 20.9, p = 0.001). The expression of CAIX correlated mildly positively with GLUT-1 (p = 0.018, rho = 0.250) as well as with Ki-67 (p = 0.014, rho = 0.295). MVD showed a significantly lower level in melanomas (mean 12.6, SD 7.7) versus sarcomas and carcinomas (mean 21.8/ 26.9, SD 13.0/20.4, p = 0.001). Median vascularity and blood volume were significantly lower in sarcomas (mean 10.4%, SD 11.0, and mean 6.3%, SD 6.5, respectively) versus carcinomas (mean 39.2%, SD 16.4 and mean 33.0%, SD 25.6, respectively) and melanomas (mean 36.0%, SD 18.3, and 31.5%, SD 24.5). Between the 3 histological groups, there was neither a significant difference in the GLUT-1 and CAIX score and continuous data, nor the Ki67 score, or polarographic oxygen measurements. GLUT-1 continuous data and Ki-67 (p<0.001, rho = 0.403), as well as Ki-67 and MVD (p = 0.029, rho = 0.228) correlated positively and a mild correlation was found between vascularity and GLUT-1 (p = 0.043, rho = 0.408). GLUT-1, CAIX, proliferative index and MVD levels were established as microenvironmental descriptors with the purpose of creating a baseline in order to follow changes seen in the tumor microenvironment after hypofractionated radiation with high doses.

## Introduction

The tumor microenvironment likely plays a role in different anticancer strategies [1]. Especially radiation therapy with high doses per fraction seems to yield better tumor control than the predictions of the radiobiological models [2, 3], and it has been suggested that co-targeting of tumor microenvironment, mainly tumor vasculature, but also enhanced tumor immunity, mediates tumor response mechanisms: Timmerman and Papiez comment that it is “*not* the technology, but rather the unique radiobiology […] that is truly special about (hypofractionated) stereotactic body radiation therapy” [3]. Thus, the usually overwhelming response of tissue irradiated with high doses of 8–30 Gy per fraction might be explained by the 5 R’s (repair, repopulation, redistribution, reoxygenation, radiosensitivity) as conventionally fractionated protocols but could also indicate an alternate response mechanism other than direct tumor cell toxicity. It must be assumed that additional tumor components (vasculature, tumor immunity) or non-tumorous stromal components may play a role—as for example by apoptotic cell death of the microvascular endothelium [2, 4, 5]. The quantification of these changes as well as the subsequent radiobiologically relevant consequences resulting from vascular damage, immune response or the bystander effect on the tumor microenvironment remains largely unknown to date.

The purpose of this study was to create a baseline in order to follow changes seen after hypofractionated radiation with high doses (e.g. 8–30Gy) that are in general applied in stereotactic radiosurgery (SRS) or stereotactic body radiation therapy (SBRT).

For this study, dogs with malignant oral tumors were chosen as a large animal model for the investigation of microenvironmental parameters as they are uniquely suited for repetitive minimally invasive and non-invasive observation during radiotherapy [6, 7]. In contrast to xenograft studies, canine tumors develop naturally and grow over long periods of time in the presence of an intact immune system, sharing similarities with human neoplasms such as inter-patient tumoral heterogeneity [8, 9]. Furthermore, clinically relevant tumor hypoxia exists in canine patients and polarographically measured pO_2_ values as well as perfusion parameters in spontaneous tumors during fractionated radiation therapy have been described previously [10–12]. In order to study the changes in tumor environment, several immunohistochemical descriptors were selected for this study. Hypoxia, the radiobiologically most relevant tumor environmental factor, leads to stabilization and activation of the hypoxia-inducible factor-1 (HIF-1). In consequence, HIF-1α protein binds to hypoxia responsive elements located in the promoter regions of genes (such as GLUT-1 (glucose transporter 1), CAIX (carbonic anhydrase 9)) whose expression has been found to closely correlate with polarographic oxygen measurements of pO_2_ [13–16]. GLUT-1 is a membrane-bound glycoprotein mediating glucose transport across the cell membrane and thereby allowing energy generation (via adenosine triphosphate (ATP) and anaerobic glycolysis) in hypoxic tumor cells that are distant to functional blood vessels. Increased expression of GLUT-1 has been found in canine histiocytic and soft tissue sarcoma, osteosarcoma, mammary carcinoma, and meningioma [17–20]. CAIX acts as a transmembrane glycoprotein expressed in tumors in response to hypoxia. Its function allows tumor cells to adapt to hypoxic stress by regulating pH and subsequently modifying the microenvironment. CAIX expression has also been described in canine mammary carcinoma, histiocytic and soft tissue sarcoma [17, 18, 21]. Microvessel density (MVD) and Ki-67 can be assessed in canine tumor tissue as a measure of vascularity and proliferation, respectively [22, 23].

The aim of this study was to establish immunohistochemical descriptors for pre-treatment tumor environment in spontaneously occurring malignant oral neoplasms. The relationship between minimally invasive oxygen measurements as well as perfusion parameters and the expression of a number of hypoxia-induced proteins was sought in order to set the baseline for further studies. The future goal is to describe the tumor microenvironment before and after hypofractionated RT with high doses per fraction.

## Materials and Methods

### Tissue Samples

Formalin-fixed, paraffin-embedded canine oral malignant tumor samples from client-owned dogs collected for diagnostic purposes were retrieved from the archives of the Institute of Veterinary Pathology, University of Zurich, Switzerland. Information about histopathologic diagnosis and malignancy, sex and age was retrieved from the clinical data chart. Questionable samples were reviewed by a pathologist. Malignant melanomas were included when one or more of the following criteria were met: mitotic index ≥ 4/10 HPF, nuclear atypia ≥ 30% of the cells, pigmentation < 50% of the cells [24].

### Immunohistochemistry

Immunohistochemical staining for Ki-67, Factor VIII and CAIX was performed in a Dako Autostainer (Dako, CH-6341 Baar), immunostaining for GLUT-1 was done using a Ventana Discovery XT automated staining system (Roche Diagnostics AG, CH-6343 Rotkreuz). Information about antibodies, pretreatment, incubation conditions and visualization are reported in Table 1. In brief, 3 μm sections were mounted on positively charged slides (Superfrost Plus), dried overnight at 37°C, deparaffinized, rehydrated and immersed for 10 min in 10% hydrogen peroxide to block endogenous peroxidase activity. Melanoma sections were bleached overnight through immersion in 20% hydrogen peroxide. For the Dako immunostaining system, antibody diluent (S2022) and wash buffer (S3006) were used; for the Roche system, antibody diluent (251–018) and reaction buffer (950–300) were applied. All antibodies have been evaluated in canine tissue in previous studies [17–20, 25–28]. Negative controls were done omitting the primary antibody. Tumor tissues were scored for immunoreactivity excluding regions with <60% neoplastic cells and areas of necrosis. All slides were scanned with a NanoZoomer 2.0-HT scanscope (Hamamatsu, CH-4500 Solothurn) and visualized using the NDP.view2 software (Hamamatsu). The relative number of positively labeled cells was determined in each sample and for all antigens as indicated below by computer-assisted manual counting by two investigators using 5–20 snapshots of randomly chosen regions of each sample taken at a 40x magnification.

**Table 1:** Antibodies and incubation conditions.

Anti-gen	Vendor	Anti-body Type	Catalogue no./Clone	Dilution, Incu-bation Condi-tions	Pre-treatment	Visuali-sation Method	Positive Control (Canine Tissues)
Ki67	Dako	mouse mAb, IgG1, kappa	M7240/MIB-1	1:50, 1 h, RT	HIER*, 20 min 98°C, EDTA buffer pH 9.0	ChemMate Kit	normal lymph node
GLUT-1	Sigma	rabbit pAb	SAB4502803	1:150, 1 h, 37°C	CC1st (EDTA buffer)	Red-Map-Kit	normal spinal cord
CaIX	Novus Biolo-gicals	rabbit pAb	23300002	1:1500, 1 h, RT	HIER*, 20 min 98°C, EDTA buffer pH 9.0	EnVision	normal stomach, intestine and liver
von Wille-brand Factor VIII	Dako	rabbit pAb	N1505	1:100, 30 min, RT	HIER*, 20 min 98°C, citrate buffer pH 7.0	ChemMate Kit	granula-tion tissue

* HIER = heat-induced epitope retrieval; for protocols carried out in the Dako Autostainer pretreatment was done separately in a steamer (Pascal S2800, Dako)

### GLUT-1, CAIX, Ki-67

The scoring system for GLUT-1 has been reported previously [17, 19] and was as following: 0 = <1% positive tumor cells, 1 = 1–50% positive tumor cells, and 2 = >50% positive tumor cells. The intensity of cellular staining was graded as well: 1 = weak positive staining and 2 = strong positive staining. The final immunoreactivity score was calculated as product of the two scores. For a product between 1 and 2, the combined final score was 1, if between 3 and 4, the combined final score was 2. For statistical analysis, also continuous data was recorded.

For CAIX, the amount of staining was recorded in a continuous manner. Furthermore, the scoring system was applied as described previously [13, 17]. In brief, the percentage of positive cells was scored as following: 0: <1% positive cells, 1: 1–30% positive cells, 2: >30% positive cells.

Also the scoring system for Ki-67 has been reported previously [26]. In short, the number of positively stained cells was determined by computer-assisted manual counting. The mean percentage of Ki-67-positive cells was determined for all fields.

### Microvessel Density

Microvessel density (MVD) was evaluated as described previously [29, 30]. Briefly, endothelial cells were stained with factor VIII and microvessels per high power field were recorded. The mean number of vessels per snapshot was determined by computer-assisted manual counting.

### Eppendorf Polarographic Oxygen Data

For a subset of the tumors from client-owned dogs, tissue hypoxia levels measured with the Eppendorf polarographic oxygen technique were available from a previous trial [7] along with tumor biopsies taken at the same time point and in the same tumor region. Formalin-fixed tissue blocks were available from the latter to evaluate the immunohistochemical markers. Owner consent had been obtained for that study, which was approved by the Animal Ethics Council of the Canton of Zurich, Switzerland. Eppendorf data consisted of multiple measurements in anesthetized dogs. Tumor oxygen partial pressure measurements were performed as previously described [10, 15] with a pO2-Histograph (Helzel Medical Systems, Kaltenkirchen, Germany). The probe was calibrated before each use, and then the needle electrode was placed into the tumor tissue under ultrasound guidance (ATL 5000, Philips Medical Systems, Zurich, Switzerland). A minimum of three different electrode tracks and a minimum of 50 recorded values were acquired. The oxygenation status of each individual tumor was described using the median pO2 and the hypoxic fractions (% of pO2 values <10 mmHg, <5 mmHg and <2.5 mmHg, respectively).

### Perfusion Data

Perfusion data was available for a subset of tumor samples as part of a previous trial [7] in client-owned dogs mentioned above. A 5- to 12-MHz linear transducer (ATL 5000, Philips AG, Zurich, Switzerland) was used to perform imaging. For contrast-enhanced power Doppler ultrasonography, settings were used as previously described [31]. Briefly, a region of interest (ROI) was drawn around the tumor boundaries and two measurements were computed for each ROI. Median of fractional area of Power Doppler (MDFAPD) was calculated as the number of colored pixels in the ROI divided by the total number of pixels in the ROI multiplied by 100. MDFAPD calculates the percentage area of the tumor occupied by blood vessels and therefore represents the vascularity index. Median color weighted fractional area of Power Doppler (MDCWFA) was used to assess perfusion and determined the mean blood volume within the tissue. MDFAPD and MDCWFA were determined by calculating the median of five images.

### Statistical Analysis

Data were coded in Excel (Microsoft^®^ Excel^®^ for Mac 2011, Version 14.3.2) and analyzed with a commercial statistical software package (IBM^®^ SPSS^®^ Statistics, Version 22). Descriptive statistics such as absolute and relative frequencies for discrete parameters (sex, CAIX score, GLUT-1 score) as well as mean and standard deviation for continuous parameters (age, GLUT-1 continuous, Ki-67 index, MVD count, Eppendorf measurements, perfusion data) were computed. As the assumption of normality was not fulfilled for CAIX continuous, median and interquartile range (IQR) were provided. The Kolmogorov-Smirnov and Shapiro-Wilk tests were used to check the validity of the normality assumption of the data. The one-way ANOVA together with the Bonferroni post-hoc test were used to disclose differences in histological parameters as well as age between different diagnosis groups. For continuous variables where the normality assumption was not fulfilled, the Kruskal-Wallis test was applied. Association between diagnosis and GLUT-1 score, CAIX score and sex was investigated by the Chi2-test. The non-parametric Spearman correlation was computed to disclose association between continuous variables. In addition, Spearman correlations were computed for each diagnosis groups separately. Results of statistical analysis with p-value <5% were interpreted as statistically significant.

## Results

Ninety-two formalin-fixed, paraffin-embedded canine oral malignant tumor samples were available. They included 32 sarcomas (34.7%), 30 carcinomas, (32.6%) and 30 malignant melanoma samples (32.6%). In 21 of the retrieved samples (sarcomas n = 9; carcinomas n = 7 and melanomas n = 5) the tissue hypoxic status determined by Eppendorf polarographic oxygen measurements was known. Perfusion data was available in 25 samples (sarcomas n = 10, carcinomas n = 8, melanomas n = 7).

### Demographic Data

Mean age of the patients was 9.4 years (range 1–15 years, SD 3.1). Twelve dogs were female, 17 were female spayed, 40 were male and 21 were male neutered. For 2 dogs, age and sex was not recorded. Age and sex was normally distributed with no significant difference between the 3 histological groups regarding sex. Patients in the melanoma group were significantly older (mean 10.8 years) than in the sarcoma group (mean 8.45 years).

### GLUT-1 Analysis

**Fig 1:**
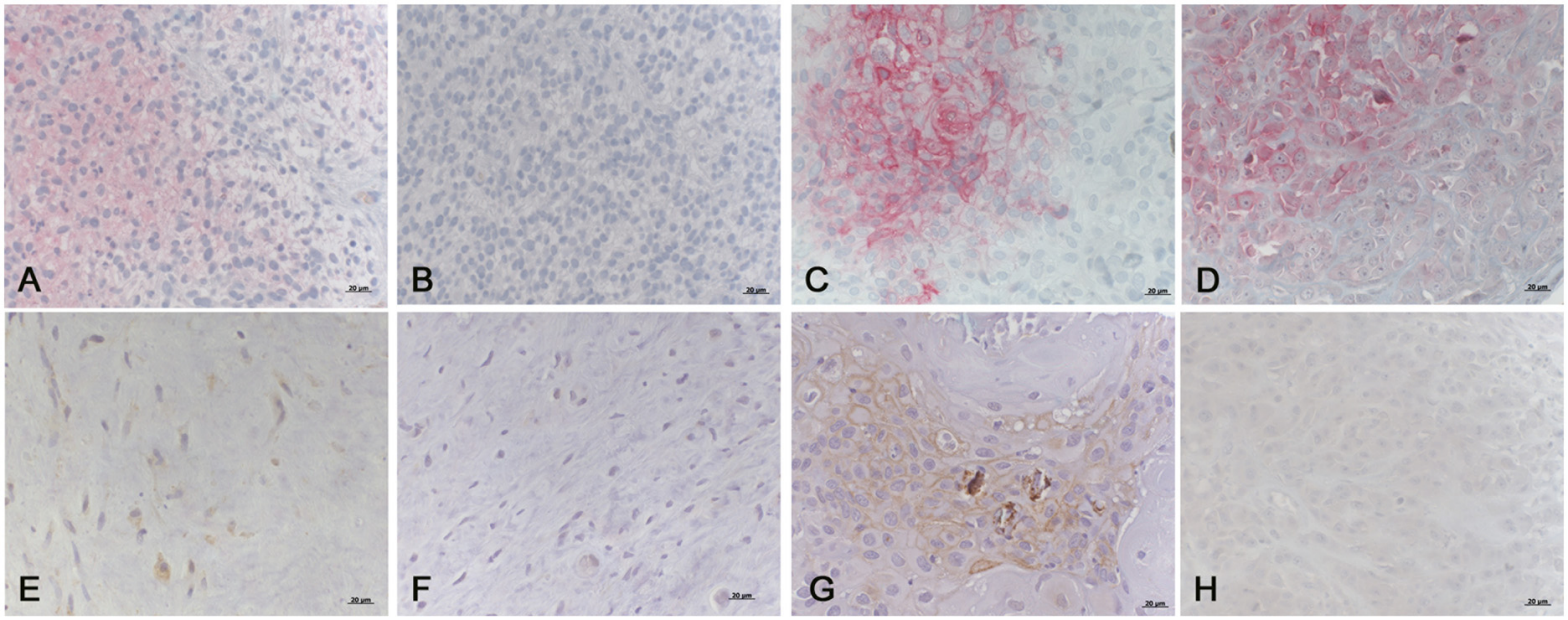
Examples of immunohistochemical labelings of canine tumors with hypoxia markers. (A) Sarcoma labelled for GLUT-1, weakly positive region. (B) Sarcoma labelled for GLUT-1, negative region, same tumor as in (A). (C) Carcinoma labelled for GLUT-1, strongly positive region. (D) Melanoma labelled for GLUT-1, strongly positive region. (E) Sarcoma labelled for CAIX, weakly positive region. (F) Sarcoma labelled for CAIX, negative region, same tumor as in (E). (G) Carcinoma labelled for CAIX, strongly positive region. (H) Melanoma labelled for CAIX, weakly positive region. Bar = 20 μm.

Immunohistochemical staining was present in 46.7% of all samples and was mainly cytoplasmatic (Fig 1). Staining intensity was weak in 76.7% and strong in 23.3% of the positive samples. The evaluation of continuous data as well as the previously described score [17] showed a significantly lower GLUT-1 level in sarcomas (mean 5.1%, SD 6.2) versus carcinomas (mean 16.5%, SD 17.3) and malignant melanomas (mean 19.0%, SD 20.9, p = 0.001) (Table 2).

**Table 2:** Immunohistochemical markers and measurements within histological groups.

Histology	GLUT-1 (%): mean, SD	CAIX (%): median, IQR	Ki-67 (%): mean, SD	MVD: mean, SD	Hypoxia (mmHg): mean, SD	Vascularity (%): mean, SD	Blood volume: mean, SD
Sarcoma (n = 32)	5.1^a^ (6.2)	2^a^ (8, range 0–76)	19.6^a^ (18.0)	21.8^b^ (13.0)	16.4^a^ (15.4)	10.4^a^ (11.9)	6.3^a^ (6.5)
Carcinoma(n = 30)	16.5^b^ (17.3)	3^a^ (20, range 0–64)	29.1^a^ (18.0)	26.9^b^ (20.4)	25.6^a^ (14.0)	39.2^b^ (16.4)	33.0^b^ (25.6)
Melanoma(n = 30)	19.0^b^ (20.9)	5^a^ (11, range 0–90)	24.9^a^ (20.4)	12.6^a^ (7.7)	24.6^a^ (40.8)	36.0^b^ (18.3)	31.5^b^ (24.5)
p-values	**0.001**	0.542	0.061	**0.001**	0.328	**0.002**	**0.002**

^a, b^: Statistically significant differences between the groups are indicated by differing letters.

### CAIX Analysis

Immunohistochemical staining was present in 34.8% of all samples (Fig 1). CAIX values were distributed in a skewed manner. There was neither a significant difference in the continuous data nor in the score between the 3 histological groups (Table 2). Overall, the expression of CAIX correlated mildly positively with GLUT-1 (p = 0.018, rho = 0.250) as well as with Ki-67 (p = 0.014, rho = 0.295). However, this correlation was lost when looking at individual histological groups.

### Ki-67 and MVD Analysis

No indication for violation of normality assumption in the data was found. For Ki67 no significant difference was found between the three histological groups, whereas MVD showed a significantly lower level in malignant melanomas (mean 12.6, SD 7.7) versus sarcomas (mean 21.8, SD 13.0) and carcinomas (mean 26.9, SD 20.4, p = 0.001) (Table 2).

### Tissue Hypoxia Levels and Perfusion Data

Eppendorf polarographic oxygen measurements did not differ significantly between the histological groups. The perfusion parameters MDFAPD and MDCWFA were significantly lower in sarcomas (mean 10.4%, SD 11.0, and mean 6.3%, SD 6.5, respectively) versus carcinomas (mean 39.2%, SD 16.4 and mean 33.0%, SD 25.6, respectively) and malignant melanomas (mean 36.0%, SD 18.3, and 31.5%, SD 24.5) (Table 2).

### Association within IHC Markers, with Polarographic Oxygen Measurements and Perfusion Data

GLUT-1 continuous data and Ki-67 (p<0.001, rho = 0.403), as well as Ki-67 and MVD (p = 0.029, rho = 0.228) correlated positively. A mild correlation was found between MDFAPD and GLUT-1 (p = 0.043, rho = 0.408). No other correlations were found.

## Discussion

In the present study, a baseline of different hypoxia-related markers and measurements in three histological groups of oral malignant neoplasia in dogs is described. The markers can be readily assessed in canine oral tumors and they do not display consistent different baseline levels according to the present study, which has been shown in human patients as well [32]. It will therefore be of great interest to evaluate the changes of such markers in individual tumors in order to gain information about processes in the tumor environment under treatment. From an ethical point of view, repetitive sampling is feasible in dog patients as they undergo a short general anesthesia for each treatment session for proper immobilization. Repetitive sampling is therefore easily achievable, once ethical approval and owner’s consent has been obtained.

The three histological groups did not show a significant difference in polarographic tissue hypoxia measurements. However, immunohistochemical markers and perfusion measurements showed small, but significant differences between the three groups with GLUT-1 levels being lowest in oral sarcoma compared to carcinoma and melanoma. CAIX and GLUT-1 showed a mild positive correlation overall, but this correlation was lost within the three histological groups. There is a direct pathophysiological link between the endogenous hypoxia markers GLUT-1 and CAIX. In the presence of hypoxia, tumors are able to and dependent on generating energy through anaerobic glycolysis. Increased glucose uptake through the cell membrane is associated with an increase of lactate and protons, i.e. with acidification. CAIX plays an important role in balancing this acidic environment by eliminating those products [33, 34]. Previous reports have shown co-expression of GLUT-1 and CAIX but their spatial distribution might be different [13, 34–36]. There is evidence that GLUT-1 expression is influenced by various factors such as glucose deprivation, oncogenic transformation, and osmotic stress, and it is unrelated to hypoxia in some cancers in humans [37–40]. CAIX seems to be less dependent on other factors, possibly rendering it a more reliable endogenous hypoxia-related marker [33, 34].

The authors are aware of the fact that the so called “endogenous hypoxia markers” (such as GLUT-1 and CAIX) as HIF-dependent products assess HIF-activity rather than the “true radiobiological relevant hypoxic fraction”. It has therefore been proposed to call them “hypoxia-related markers” [33]. The oxygen concentration required to stabilize and activate HIF is in the range of 1–2%, while the concentration causing maximum resistance to radiation is much lower with about 0.02%. In some genetic alterations, also hypoxia-independent regulation of HIF can occur [41]. However, evaluation of the transcriptional targets of HIF reflects part of the changes of tumor environment over a course of treatment and can be followed in repetitive samples.

Neither of the endogenous hypoxia-related markers GLUT-1 and CAIX correlated with polarographic tissue hypoxia measurements in the present study. In general, the association between the oxygen status of tumor tissue and the hypoxia-related markers is described to be weak and none of the markers have shown consistent strong prognostic impact in the clinical setting [33]. Previous studies in human cancer patients showed inconsistent results: While some studies showed a positive correlation between tissue oxygen measurements and endogenous hypoxia markers GLUT-1 and CAIX [14, 42], others failed to find any association [16, 36, 43]. A possible explanation is that readouts of polarographic oxygen content of tissues cannot distinguish between areas with necrosis and viable tissue, and can therefore possibly lead to an overestimation of hypoxia. In contrast, endogenous hypoxia-related markers can be detected in hypoxic areas with viable cells only [42] and are found already at higher levels of tissue oxygenation. While polarographic measurements evaluate tissue oxygen status directly, GLUT-1 and CAIX are markers for hypoxia response pathways caused by the presence of low oxygen levels and other factors. Increase of GLUT-1 is a sign of energy generation by anaerobic glycolysis due to hypoxia-induced ATP depletion, while CAIX is responsible for pH regulation in a hypoxic, acidic environment [14, 33, 42]. This response might already take place well above the level of hypoxia detected by polarographic Eppendorf measurements [34]. It is also possible that the site of the biopsy samples may not have been respresentative of the areas sampled for tissue oxygenation measurements. Tumor hypoxia is a dynamic process, and abnormal vasculature might lead to transient changes in tumoral blood flow, with hypoxic or well-oxygenated conditions changing over time. Those spatially and temporally fluctuating conditions might lead to a discrepancy in measurements. Furthermore, prognostic factors such as tumor size, location, histological subtype, and grade of differentiation were not taken into account due to lack of information and/or due to small numbers per individual group. It should be recognized that pooling of histologic subtypes via crude classification may preclude detection of statistically significant differences due to elevated type II error. This represents an important limitation of the present study. The influence on prognosis of the aforementioned factors is most probably dependent on the treatment modality and the ability of achieving adequate local control. This might be more challenging in in maxillary tumors, caudal location or tumors with a large size [44–47]. Due to the retrospective nature of this study, information about treatment and outcome could not be gathered. This represents a limitation of this study but could not be avoided herein.

Tumor perfusion has been found to moderately correlate with MVD in canine tumors but no significant change was seen during the course of a curative-intent RT protocol in a small number of patients [31, 48]. Our findings indicate that sarcomas are least perfused in spite of having a higher MVD than malignant melanomas for instance. The mere presence of vessels does not necessarily guarantee physiological functionality thereof, which may also depend on diameter, architecture or intratumoral pressure.

Small (repetitive) tumor sampling bears the risk of a lack of representation of the whole tumor due to intratumoral heterogeneity in the extent and distribution of malignancy, stromal reaction, inflammatory cells, necrosis, and hypoxia. However, in a clinical setting it is not feasible to collect larger or multiple biopsy samples either concurrently or repetitively. With the outlook of repetitive sampling during hypofractionated stereotactic fractionation in clinical patients in the future, a power analysis was performed to test the applicability for such a further question in a presumably rather small clinical patient cohort. In order to detect a relevant difference with potential clinical impact of 25% with GLUT-1 and CAIX, 30% with Ki-67, and 30% with MVD between two histological groups with the two sample t-test given power of 80% and a Bonferroni corrected significance level of alpha = 0.016 the optimal sample size of 10, 14, and 17 observations in each diagnosis group would be required. If the assumptions above are correct, the sample sizes of 32, 30, and 30 tumor samples in the sarcoma, carcinoma, and malignant melanoma groups should therefore guarantee correct detection of relevant differences that we would deem clinically relevant with probability exceeding 80%. Consequently, there is some indication for no variation of the baseline levels in the population evaluated.

A previous study in dogs sequentially evaluating hypoxia-related markers and tumor/ microenvironmental factors during RT showed inconsistent results [6]. It remains to be evaluated if there are marked differences between those parameters during conventional fractionation versus hypofractionated treatment protocols, as such fractionation schemes are increasingly applied for example in SRS and SBRT, even though the knowledge about the exact mechanisms of action remains small at this point.

## Supporting Information

S1 Table Demographic data of study cases. (DOCX)

S2 Table Hypoxia-related markers and microenvironmental descriptors. (DOCX)

S3 Table Possible prognostic factors of study cases. (DOCX)
