# Supplementary material for: Hypoxia-Related Marker GLUT-1, CAIX, Proliferative Index and Microvessel Density in Canine Oral Malignant Neoplasia
Source: PLoS One. 2016 Feb 23;11(2):e0149993. doi: 10.1371/journal.pone.0149993 (PMC4764341; doi:10.1371/journal.pone.0149993)
Supplement: S1 Table — (DOCX) [file pone.0149993.s001.docx]

S1 Table. Demographic data of study cases.

| **Case Number** | **Diagnosis Code**  (0: oral sarcoma; 1: oral carcinoma;  2: oral malignant melanoma) | **Tumor Type** | **Age**  (years) | **Gender Code**  (1: male; 2: male neutered; 3: female; 4: female spayed) | **Breed** |
| --- | --- | --- | --- | --- | --- |
| 1 | 0 | FSA | 9 | 1 | Mixed Breed |
| 2 | 0 | FSA | 9 | 3 | Mixed Breed |
| 3 | 0 | FSA | 5 | 2 | Bernese Mountain Dog |
| 4 | 0 | FSA | 11 | 4 | Mixed Breed |
| 5 | 0 | spindle-cell sarcoma | 15 | 1 | Mixed Breed |
| 6 | 0 | FSA | 10 | 2 | Golden Retriever |
| 7 | 0 | FSA | 7 | 1 | Rottweiler |
| 8 | 0 | FSA | 8 | 1 | Rhodesian Ridgeback |
| 9 | 0 | FSA | 8 | 2 | Collie |
| 10 | 0 | FSA | 7 | 4 | Mixed Breed |
| 11 | 0 | FSA | 10 | 2 | Labrador Retriever |
| 12 | 0 | FSA | 11 | 4 | Mixed Breed |
| 13 | 0 | myxosarcoma | 7 | 4 | Pitbull Terrier |
| 14 | 0 | FSA | 6 | 1 | Great Dane |
| 15 | 0 | sarcoma | 11 | 1 | Boxer |
| 16 | 0 | FSA | 2 | 1 | Magyar Vizsla |
| 17 | 0 | FSA | 13 | 1 | Cocker Spaniel |
| 18 | 0 | FSA | 6 | 1 | Leonberger |
| 19 | 0 | chondrosarcoma | 10 | 1 | Mixed Breed |
| 20 | 0 | myxosarcoma | 9 | 2 | Mixed Breed |
| 21 | 0 | spindle-cell sarcoma | 10 | 1 | Neufoundland |
| 22 | 0 | spindle-cell sarcoma | 7 | 1 | Flat Coated Retriever |
| 23 | 0 | spindle-cell sarcoma | 7 | 1 | Tibetan Terrier |
| 24 | 0 | spindle-cell sarcoma | 7 | 4 | Mixed Breed |
| 25 | 0 | FSA | 2.5 | 4 | Boxer |
| 26 | 0 | sarcoma | N/A | 2 | Eurasian Dog |
| 27 | 0 | osteosarcoma | 5 | 3 | Parson Jack Russel |
| 28 | 0 | fibromyxosarcoma | 9 | 4 | Swiss White Shepherd |
| 29 | 0 | osteosarcoma | 9 | 1 | Mixed Breed |
| 30 | 0 | sarcoma | 12 | 2 | Giant Schnauzer |
| 31 | 0 | osteosarcoma | 11 | 2 | American Staffordshire Terrier |
| 32 | 0 | FSA | N/A | N/A | N/A |
| 33 | 1 | non-keratizing SCC | 15 | 3 | Yorkshire Terrier |
| 34 | 1 | SCC | 9 | 2 | Mixed Breed |
| 35 | 1 | SCC | 12 | 3 | Yorkshire Terrier |
| 36 | 1 | basal cell carcinoma | 6 | 3 | Collie |
| 37 | 1 | non-keratizing SCC | 11 | 1 | Newfoundland |
| 38 | 1 | SCC | 5 | 1 | Bernese Mountain Dog |
| 39 | 1 | SCC | 11 | 1 | Golden Retriever |
| 40 | 1 | keratizing SCC | 12 | 3 | West Highland White Terrier |
| 41 | 1 | non-keratizing SCC | 5 | 3 | Boxer |
| 42 | 1 | SCC | 7 | 3 | Golden Retriever |
| 43 | 1 | SCC | 10 | 2 | Golden Retriever |
| 44 | 1 | scirrhous carcinoma | 12 | 3 | Cocker Spaniel |
| 45 | 1 | SCC | 8 | 1 | Mixed Breed |
| 46 | 1 | anaplastic carcinoma | 11 | 4 | Golden Retriever |
| 47 | 1 | SCC | 4 | 3 | West Highland White Terrier |
| 48 | 1 | non-keratizing SCC | 1 | 2 | Labrador Retriever |
| 49 | 1 | SCC | 11 | 1 | Mixed Breed |
| 50 | 1 | SCC | 7 | 1 | Golden Retriever |
| 51 | 1 | keratizing SCC | 10 | 1 | Entlebucher Mountain Dog |
| 52 | 1 | keratizing SCC | 8 | 2 | Mixed Breed |
| 53 | 1 | keratizing SCC | 12 | 1 | Cairn Terrier |
| 54 | 1 | SCC | 13 | 4 | Miniature Schnauzer |
| 55 | 1 | anaplastic carcinoma | 10 | 1 | Bergamasco Shepherd |
| 56 | 1 | SCC | 6 | 1 | Labrador Retriever |
| 57 | 1 | SCC | 11 | 2 | Collie |
| 58 | 1 | SCC | 7 | 1 | Lhaso Apso |
| 59 | 1 | SCC | 9 | 4 | Giant Schnauzer |
| 60 | 1 | SCC | 5 | 4 | Australian Shepherd |
| 61 | 1 | SCC | 13 | 4 | Mixed Breed |
| 62 | 1 | SCC | 10 | 3 | Yorkshire Terrier |
| 63 | 2 | MM | 11 | 1 | Dachshund |
| 64 | 2 | MM | 12 | 1 | Bernese Mountain Dog |
| 65 | 2 | MM | 8 | 1 | Standard Poodle |
| 66 | 2 | MM | 12 | 1 | Golden Retriever |
| 67 | 2 | MM | 5 | 2 | Mixed Breed |
| 68 | 2 | MM | 11 | N/A | Bernese Mountain Dog |
| 69 | 2 | MM | 13 | 2 | Mixed Breed |
| 70 | 2 | MM | 12 | 4 | Mixed Breed |
| 71 | 2 | amelanotic MM | 8 | 4 | Airdale Terrier |
| 72 | 2 | MM | 6 | 1 | Mixed Breed |
| 73 | 2 | MM | 13 | 1 | Cocker Spaniel |
| 74 | 2 | MM | 11 | 1 | Mixed Breed |
| 75 | 2 | MM | 9 | 2 | Staffordshire Bull Terrier |
| 76 | 2 | amelanotic MM | 8 | 2 | Miniature Poodle |
| 77 | 2 | amelanotic MM | 12 | 1 | West Highland White Terrier |
| 78 | 2 | MM | 14 | 1 | Mixed Breed |
| 79 | 2 | amelanotic MM | 13 | 4 | Yorkshire Terrier |
| 80 | 2 | amelanotic MM | 14 | 1 | Pekingese |
| 81 | 2 | MM | 12 | 1 | Cocker Spaniel |
| 82 | 2 | MM | 15 | 4 | German Shepherd |
| 83 | 2 | MM | 11 | 2 | Labrador Retriever |
| 84 | 2 | MM | 11 | 2 | Labrador Retriever |
| 85 | 2 | MM | 8 | 1 | Magyar Vizsla |
| 86 | 2 | amelanotic MM | 7 | 3 | Kromfohrlander |
| 87 | 2 | MM | 13 | 1 | Munsterlander |
| 88 | 2 | MM | 14 | 4 | Poodle |
| 89 | 2 | MM | 12 | 2 | Mixed Breed |
| 90 | 2 | MM | 11 | 1 | Dachshund |
| 91 | 2 | MM | 6 | 1 | Mixed Breed |
| 92 | 2 | MM | 12 | 2 | Doberman Pinscher |

N/A: not applicable (no information available); FSA: fibrosarcoma, MM: malignant melanoma; SCC: squamous cell carcinoma
