# Supplementary material for: Hypoxia-Related Marker GLUT-1, CAIX, Proliferative Index and Microvessel Density in Canine Oral Malignant Neoplasia
Source: PLoS One. 2016 Feb 23;11(2):e0149993. doi: 10.1371/journal.pone.0149993 (PMC4764341; doi:10.1371/journal.pone.0149993)
Supplement: S2 Table — (DOCX) [file pone.0149993.s002.docx]

S2 Table. Hypoxia-related markers and microenvironmental descriptors.

| **Case Number** | **GLUT-1 Continuous Data**  (%) | **GLUT-1 Score**  (0: <1% positive; 1: 1-50% positive; 2: >50% positive) | **GLUT-1 Intensity** (1: weak; 2: strong) | **GLUT-1 Final Score** (score x intensity; 1: 1-2; 2: 3-4) | **Ki-67 Score** (%) | **MVD Score** | **CAIX Score**  (0: <1% positive cells, 1: 1-30% positive cells, 2: >30% positive cells | **CAIX Continuous Data** | **MDFAPD**  (%) | **MDCWFA**  (%) | **EPPMD**  (mmHg) |
| --- | --- | --- | --- | --- | --- | --- | --- | --- | --- | --- | --- |
| 1 | 1 | 0 | 0 | 0 | 8 | 22 | 0 | 0 | 1.97 | 1.08 | 0.15 |
| 2 | 9 | 0 | 0 | 0 | 6 | 30 | 0 | 0 | 18.363 | 13.233 |  |
| 3 | 5 | 1 | 1 | 1 | 3 | 37 | 0 | 0 | 7.09 | 3.82 | 5.30 |
| 4 | 21 | 1 | 1 | 1 | 26 | 23 |  |  | 2.57 | 2.06 | 5.20 |
| 5 | 6 | 0 | 0 | 0 | 34 | 11 | 0 | 0 | 17.54 | 12.71 | 43.10 |
| 6 | 3 | 0 | 0 | 0 | 11 | 20 | 1 | 15 |  |  |  |
| 7 | 1 | 0 | 0 | 0 | 16 | 17 | 0 | 0 | 0.11 | 0.02 | 10.79 |
| 8 | 1 | 0 | 0 | 0 | 5 | 14 |  |  |  |  |  |
| 9 | 0 | 0 | 0 | 0 | 2 | 38 | 0 | 5 | 5 | 3.17 |  |
| 10 | 0 | 0 | 0 | 0 | 8 | 36 | 0 | 8 | 11.12 | 6.59 | 6.90 |
| 11 | 3 | 0 | 0 | 0 | 16 | 18 | 0 | 1 |  |  |  |
| 12 | 0 | 0 | 0 | 0 | 1 | 12 | 0 | 0 |  |  | 36.51 |
| 13 | 3 | 0 | 0 | 0 | 18 | 11 | 0 | 0 | 38.788 | 19.078 | 27.92 |
| 14 | 9 | 0 | 0 | 0 | 5 | 22 | 0 | 0 |  |  |  |
| 15 | 4 | 0 | 0 | 0 | 16 | 21 | 1 | 8 |  |  |  |
| 16 | 0 | 0 | 0 | 0 | 13 | 16 | 0 | 2 |  |  |  |
| 17 | 4 | 0 | 0 | 0 | 14 | 16 | 0 | 1 |  |  |  |
| 18 | 0 | 0 | 0 | 0 | 9 | 19 | 0 | 2 |  |  |  |
| 19 | 21 | 1 | 2 | 1 | 41 | 74 | 1 | 13 |  |  |  |
| 20 | 6 | 0 | 0 | 0 | 25 | 6 | 0 | 1 |  |  |  |
| 21 | 14 | 1 | 1 | 1 | 29 | 20 | 0 | 4 |  |  |  |
| 22 | 1 | 0 | 0 | 0 | 10 | 17 | 0 | 3 |  |  |  |
| 23 | 17 | 1 | 1 | 1 | 9 | 27 | 0 | 6 |  |  |  |
| 24 | 3 | 0 | 0 | 0 | 27 | 26 | 1 | 49 |  |  |  |
| 25 | 6 | 0 | 0 | 0 | 18 | 38 | 0 | 0 |  |  |  |
| 26 | 10 | 0 | 0 | 0 | 83 | 15 | 2 | 76 |  |  |  |
| 27 | 2 | 0 | 0 | 0 | 51 | 21 | 0 | 9 |  |  |  |
| 28 | 0 | 0 | 0 | 0 | 54 | 13 | 1 | 27 |  |  |  |
| 29 | 14 | 0 | 0 | 0 | 9 | 9 | 1 | 17 |  |  |  |
| 30 | 0 | 0 | 0 | 0 | 27 | 30 | 0 | 0 |  |  |  |
| 31 | 0 | 0 | 0 | 0 | 32 | 8 | 1 | 7 |  |  |  |
| 32 | 0 | 0 | 0 | 0 | 0 | 11 | 0 | 0 | 1.537 | 0.897 | 11.45 |
| 33 | 3 | 0 | 0 | 0 | 17 | 18 | 2 | 36 |  |  |  |
| 34 | 12 | 1 | 1 | 1 | 43 | 11 | 0 | 0 |  |  |  |
| 35 | 35 | 1 | 1 | 1 | 29 | 24 | 0 | 0 |  |  |  |
| 36 | 11 | 1 | 1 | 1 | 8 | 9 | 0 | 1 |  |  |  |
| 37 | 25 | 1 | 2 | 1 | 22 | 9 | 0 | 3 |  |  |  |
| 38 | 25 | 1 | 1 | 1 | 17 | 32 | 1 | 3 |  |  |  |
| 39 | 8 | 0 | 0 | 0 | 34 | 9 | 1 | 10 |  |  |  |
| 40 | 6 | 0 | 0 | 0 | 8 | 8 | 0 | 1 |  |  |  |
| 41 | 0 | 0 | 0 | 0 | 41 | 6 | 0 | 1 |  |  |  |
| 42 | 2 | 0 | 0 | 0 | 23 | 12 | 0 | 1 | 30.953 | 29.245 | 48.30 |
| 43 | 5 | 1 | 1 | 1 | 57 | 87 | 0 | 0 | 47.84 | 26.81 | 22.20 |
| 44 | 14 | 1 | 1 | 1 | 20 | 13 | 0 | 0 |  |  |  |
| 45 | 17 | 1 | 2 | 1 | 12 | 56 | 0 | 0 | 53.47 | 23.71 | 19.97 |
| 46 | 27 | 1 | 1 | 1 | 62 | 35 | 1 | 9 | 14.33 | 8.66 | 31.19 |
| 47 | 23 | 1 | 1 | 1 | 23 | 56 | 1 | 6 | 21.89 | 11.15 |  |
| 48 | 6 | 0 | 0 | 0 | 11 | 41 | 1 | 21 | 62.12 | 86.08 |  |
| 49 | 20 | 1 | 1 | 1 | 46 | 74 | 1 | 9 |  |  | 36.00 |
| 50 | 7 | 1 | 1 | 1 | 16 | 33 | 0 | 1 | 48.15 | 54.58 | 15.04 |
| 51 | 3 | 0 | 0 | 0 | 25 | 24 |  |  |  |  |  |
| 52 | 46 | 1 | 2 | 1 | 45 | 22 | 1 | 25 |  |  |  |
| 53 | 29 | 1 | 2 | 1 | 35 | 31 | 1 | 25 |  |  |  |
| 54 | 0 | 0 | 0 | 0 | 8 | 17 | 0 | 1 |  |  |  |
| 55 | 1 | 0 | 0 | 0 | 18 | 9 | 1 | 12 |  |  |  |
| 56 | 14 | 1 | 1 | 1 | 42 | 34 | 1 | 27 |  |  |  |
| 57 | 0 | 0 | 0 | 0 | 75 | 25 | 0 | 0 |  |  |  |
| 58 | 0 | 0 | 0 | 0 | 0 | 9 | 0 | 0 |  |  |  |
| 59 | 48 | 2 | 2 | 2 | 35 | 51 | 1 | 32 |  |  |  |
| 60 | 1 | 0 | 0 | 0 | 34 | 14 | 2 | 64 |  |  |  |
| 61 | 69 | 2 | 2 | 2 | 51 | 20 | 1 | 10 |  |  |  |
| 62 | 39 | 1 | 1 | 1 | 16 | 18 | 1 | 19 | 35.24 | 23.93 | 6.39 |
| 63 | 74 | 2 | 1 | 1 | 41 | 25 | 0 | 16 |  |  |  |
| 64 | 62 | 2 | 1 | 1 | 37 | 27 | 0 | 1 |  |  |  |
| 65 | 1 | 0 | 0 | 0 | 11 | 13 | 0 | 0 | 44.636 | 38.974 | 2.50 |
| 66 | 2 | 0 | 0 | 0 | 66 | 25 | 0 | 4 | 16.15 | 8.8 | 0.00 |
| 67 | 7 | 0 | 0 | 0 | 6 | 9 | 0 | 5 | 27.23 | 15.24 |  |
| 68 | 15 | 1 | 1 | 1 | 23 | 19 | 1 | 39 |  |  |  |
| 69 | 74 | 2 | 1 | 1 | 36 | 6 | 0 | 0 |  |  |  |
| 70 | 17 | 1 | 1 | 1 | 17 | 6 | 0 | 0 | 44.66 | 34.92 |  |
| 71 | 1 | 0 | 0 | 0 | 3 | 3 | 0 | 0 |  |  |  |
| 72 | 14 | 1 | 1 | 1 | 64 | 16 | 0 | 0 | 68.651 | 81.421 | 95.38 |
| 73 | 13 | 1 | 1 | 1 | 1 | 12 | 0 | 0 |  |  |  |
| 74 | 17 | 1 | 1 | 1 | 15 | 9 | 0 | 1 |  |  |  |
| 75 | 13 | 1 | 1 | 1 | 31 | 5 | 0 | 4 |  |  |  |
| 76 | 15 | 1 | 2 | 1 | 12 | 13 | 2 | 90 |  |  |  |
| 77 | 23 | 1 | 1 | 1 | 31 | 12 | 0 | 1 |  |  |  |
| 78 | 1 | 0 | 0 | 0 | 4 | 4 | 0 | 6 |  |  |  |
| 79 | 12 | 1 | 1 | 1 | 33 | 20 | 0 | 5 |  |  |  |
| 80 | 33 | 1 | 1 | 1 | 68 | 31 | 0 | 6 |  |  |  |
| 81 | 3 | 0 | 0 | 0 | 36 | 17 | 0 | 0 |  |  |  |
| 82 | 16 | 1 | 1 | 1 | 9 | 6 | 1 | 28 |  |  |  |
| 83 | 23 | 1 | 2 | 1 | 63 | 10 | 1 | 24 |  |  |  |
| 84 | 16 | 1 | 1 | 1 | 27 | 17 | 1 | 8 |  |  |  |
| 85 | 0 | 0 | 0 | 0 | 5 | 11 | 1 | 15 |  |  |  |
| 86 | 49 | 1 | 1 | 1 | 33 | 6 | 2 | 43 |  |  |  |
| 87 | 0 | 0 | 0 | 0 | 8 | 14 | 0 | 7 |  |  |  |
| 88 | 21 | 1 | 2 | 1 | 30 | 11 | 0 | 7 |  |  |  |
| 89 | 3 | 0 | 0 | 0 | 4 | 19 | 1 | 9 |  |  |  |
| 90 | 36 | 1 | 1 | 1 | 7 | 3 | 0 | 6 | 32 | 24.3 | 1.20 |
| 91 | 0 | 0 | 0 | 0 | 0 | 2 | 0 | 0 | 18.7 | 16.77 | 23.90 |
| 92 | 8 | 1 | 1 | 1 | 25 | 6 | 0 | 0 |  |  |  |

GLUT-1: glucose transporter-1; MVD: microvessel density; CAIX: carbonic anhydrase IX; MDFAPD: median of fractional area of Power Doppler; MDCWFA: median color weighted fractional area of Power Doppler; EPPMD: median polarographic Eppendorf pO2
