# Supplementary material for: Hypoxia-Related Marker GLUT-1, CAIX, Proliferative Index and Microvessel Density in Canine Oral Malignant Neoplasia
Source: PLoS One. 2016 Feb 23;11(2):e0149993. doi: 10.1371/journal.pone.0149993 (PMC4764341; doi:10.1371/journal.pone.0149993)
Supplement: S3 Table — (DOCX) [file pone.0149993.s003.docx]

S3 Table. Possible prognostic factors of study cases.

| **Case Number** | **Diagnosis Code**  (0: oral sarcoma; 1: oral carcinoma;  2: oral malignant melanoma) | **Tumor Grade** | **Location Jaw**  (1: mandible; 2: maxilla) | **Location Head**  (1: rostral; 2: caudal) | **Mitotic Index**  (1: low; 2: high) | **Differentiation**  (1: highly differentiated; 2: poorly differentiated) | **Tumor Size**  (longest diameter; cm) |
| --- | --- | --- | --- | --- | --- | --- | --- |
| 1 | 0 | 1 | 1 | N/A | 1 | 1 |  |
| 2 | 0 | 2 | N/A | N/A | 1 | 1-2 |  |
| 3 | 0 | 1 | 2 | 2 | 1 | 1 | 5.5 |
| 4 | 0 | 1 | 2 | 1 |  | 1 | 2.9 |
| 5 | 0 |  | 2 | 1 |  |  |  |
| 6 | 0 |  | 2 | N/A | 1 |  |  |
| 7 | 0 | 1 | 2 | 2 | 1 | 1 |  |
| 8 | 0 | 1 | 1 | 2 | 1 | 1 |  |
| 9 | 0 | 1 | 2 | N/A |  | 1 |  |
| 10 | 0 | 2-3 | 1 | 1 | 2 | 2 |  |
| 11 | 0 |  | 2 | 2 |  |  |  |
| 12 | 0 | 1 | 2 | 1 |  | 1 |  |
| 13 | 0 |  | 2 | 1 | 1 |  | 6.3 |
| 14 | 0 | 1 | 2 | 1 | 1 | 1 | 7.6 |
| 15 | 0 |  | 2 | 1 | 1 |  | 3. |
| 16 | 0 | 1-2 | 2 | 2 | 1 | 1-2 |  |
| 17 | 0 | 2 | 2 | 2 | 1 | 2 |  |
| 18 | 0 | 1 | 2 | 1 | 1 | 1 | 5 |
| 19 | 0 |  | 1 | 2 | 1 |  | >2 |
| 20 | 0 |  | 2 | 2 | 2 |  | 5 |
| 21 | 0 |  | 1 | 2 |  |  | 3 |
| 22 | 0 |  | 2 | 1 |  |  |  |
| 23 | 0 |  | 2 | 2 |  |  | 5 |
| 24 | 0 |  | 2 | 2 | 1 |  |  |
| 25 | 0 |  | 2 | 2 | 2 |  |  |
| 26 | 0 |  | 2 | 2 | 2 |  |  |
| 27 | 0 |  | 2 | 2 | 2 |  |  |
| 28 | 0 |  | 2 | 2 | 1 |  |  |
| 29 | 0 |  | 2 | 1 |  |  |  |
| 30 | 0 |  | 2 | 1 | 2 |  | 2 |
| 31 | 0 |  | 1 | 2 | 2 |  |  |
| 32 | 0 |  | 2 | 1-2 | 1 | 1 | 3 |
| 33 | 1 |  | 1 | N/A | 2 | 1-2 |  |
| 34 | 1 |  | 1 | N/A |  |  |  |
| 35 | 1 |  | 1 | N/A | 2 |  |  |
| 36 | 1 |  | N/A | N/A | 1 |  |  |
| 37 | 1 |  | N/A | N/A |  |  |  |
| 38 | 1 |  | N/A | N/A |  | 1 |  |
| 39 | 1 | 1 | 1 | 1 |  | 1 | 3 |
| 40 | 1 |  | 1 | 1 | 1 |  | 2.4 |
| 41 | 1 |  | 1 | N/A | 2 |  |  |
| 42 | 1 | 1 | 1 | 2 |  |  | 6 |
| 43 | 1 |  | N/A | N/A | 2 | 2 |  |
| 44 | 1 |  | 1 | 1 |  |  |  |
| 45 | 1 |  | 1 | 1 |  | 1 |  |
| 46 | 1 |  | 1 | 2 | 1 |  | 6 |
| 47 | 1 |  | 2 | 2 | 1 |  | 5.6 |
| 48 | 1 |  | 1 | 1 | 1-2 |  |  |
| 49 | 1 |  | 2 | N/A | 1 |  |  |
| 50 | 1 |  | 1 | 1 | 1-2 | 1 |  |
| 51 | 1 |  | 2 | 2 |  |  |  |
| 52 | 1 |  | 2 | 2 | 2 |  | 4 |
| 53 | 1 |  | 2 | 2 | 1-2 |  | 3.3 |
| 54 | 1 |  | 2 | 1 | 2 |  |  |
| 55 | 1 |  | 1 | 2 |  |  | 4 |
| 56 | 1 |  | 1 | 1 | 2 |  |  |
| 57 | 1 |  | 2 | 1 |  |  |  |
| 58 | 1 |  | 2 | 1 |  | 1 |  |
| 59 | 1 |  | 2 | 1 | 1 |  |  |
| 60 | 1 |  | 1 | 1 |  |  |  |
| 61 | 1 |  | 1 | 2 | 1 |  | 3 |
| 62 | 1 |  | 1 | 2 | 1-2 |  | 4 |
| 63 | 2 |  | N/A | N/A | 2 |  |  |
| 64 | 2 |  | 2 | 2 | 2 |  | 4 |
| 65 | 2 |  | N/A | N/A | 2 |  |  |
| 66 | 2 |  | 1 | N/A | 2 |  |  |
| 67 | 2 |  | 1 | 2 |  |  | 0.5 |
| 68 | 2 |  | 2 | N/A | 2 |  |  |
| 69 | 2 |  | 2 | 2 | 2 |  |  |
| 70 | 2 |  | 1 | 1 | 2 |  |  |
| 71 | 2 |  | 2 | N/A |  |  |  |
| 72 | 2 |  | 2 | 2 |  |  | 2.6 |
| 73 | 2 |  | 1 | N/A |  |  |  |
| 74 | 2 |  | 2 | N/A | 1 |  | 2 |
| 75 | 2 |  | 1 | 2 |  |  | 2.5 |
| 76 | 2 |  | 1 | 1 | 2 |  |  |
| 77 | 2 |  | 2 | 2 | 2 |  |  |
| 78 | 2 |  | 2 | 1 | 2 | 1 | 3.3 |
| 79 | 2 |  | 2 | N/A | 2 |  |  |
| 80 | 2 |  | 2 | 1 | 2 |  | 3.5 |
| 81 | 2 |  | 2 | 2 | 1-2 |  |  |
| 82 | 2 |  | 1 | 1 | 2 |  |  |
| 83 | 2 |  | 2 | 1 | 1-2 |  | 3 |
| 84 | 2 |  | 2 | 1 |  |  | 2.6 |
| 85 | 2 |  | 1 | 1 | 1 |  | 1 |
| 86 | 2 |  | 2 | 2 | 1-2 |  |  |
| 87 | 2 |  | 1 | 1 | 1-2 |  |  |
| 88 | 2 |  | 2 | 2 | 2 |  |  |
| 89 | 2 |  | 2 | 1 | 1 |  | 1 |
| 90 | 2 |  | 2 | N/A | 1 |  | 0.5 |
| 91 | 2 |  | 2 | 2 | 2 |  | 6 |
| 92 | 2 |  | 1 | 2 |  |  | 3 |

N/A: not applicable (no information available)
